# Comparative Evaluation of Depth of Irrigant Penetration Using Different Irrigation Systems: An In Vivo Study

**DOI:** 10.7759/cureus.114841

**Published:** 2026-08-20

**Authors:** Akshata Ohm, Rishi Manan, Priyanka Mishra, Bharat Chauhan, Ajay B Gutti, Arjun Soni

**Affiliations:** 1 Department of Conservative Dentistry and Endodontics, Institute of Dental Studies and Technologies, Modinagar, IND; 2 Department of Conservative Dentistry and Endodontics, Malla Reddy Institute of Dental Sciences, Hyderabad, IND

**Keywords:** diode laser irrigation, irrigant penetration, mandibular molars, root canal irrigation, sonic activation

## Abstract

Introduction: Effective irrigation is essential for successful root canal treatment because mechanical instrumentation alone cannot adequately clean the complex anatomy of the root canal system. Various irrigant activation techniques have been developed to enhance irrigant penetration into the apical third and anatomically inaccessible regions; however, clinical evidence comparing their penetration efficiency remains limited. The aim of our study was to compare the depth of irrigant penetration achieved using conventional manual irrigation, passive ultrasonic irrigation, sonic irrigation, air-sonic irrigation, and diode laser-assisted irrigation in the mesial canals of mandibular first molars.

Materials and methods: This in vivo study included 50 mandibular first molars requiring primary root canal treatment. Following standardized biomechanical preparation, the teeth were managed using one of five irrigation activation techniques: conventional manual irrigation, passive ultrasonic irrigation, sonic irrigation, air-sonic irrigation (PATS Vario; Innovations Endo, Nashik, India), or diode laser-assisted irrigation (n = 10 per group). Iohexol contrast medium was used to evaluate irrigant penetration, and standardized digital radiographs were obtained. The linear distance between the working length and the maximum extent of dye penetration was measured using dedicated imaging software. Data were analyzed using one-way analysis of variance followed by Tukey's post hoc test, with the level of statistical significance set at p < 0.05.

Results: Irrigant penetration differed significantly among the five irrigation activation techniques (p < 0.001, η² = 0.55). Diode laser-assisted irrigation showed the greatest apical penetration (0.33 ± 0.26 mm), followed by sonic irrigation (0.72 ± 0.42 mm), PATS air-sonic irrigation (0.87 ± 0.59 mm), passive ultrasonic irrigation (1.23 ± 0.78 mm), and conventional manual irrigation (2.27 ± 0.88 mm). Pairwise analysis demonstrated significantly better penetration with diode laser-assisted, sonic, PATS air-sonic, and passive ultrasonic irrigation than with conventional manual irrigation (all p < 0.05). Diode laser-assisted irrigation also outperformed passive ultrasonic irrigation (p = 0.028), whereas no significant differences were observed between diode laser-assisted irrigation and sonic or PATS air-sonic irrigation, or among the sonic, PATS air-sonic, and passive ultrasonic irrigation groups (all p > 0.05).

Conclusion: Irrigant activation significantly improved apical irrigant penetration compared with conventional manual irrigation. Among the evaluated techniques, diode laser-assisted irrigation demonstrated the greatest penetration efficiency, suggesting its potential to enhance canal disinfection in clinical endodontic practice. Sonic and PATS air-sonic irrigation also provided favorable penetration and may serve as effective alternatives when laser systems are unavailable.

## Introduction

Successful root canal treatment depends on effective cleaning and disinfection of the entire root canal system. Although mechanical instrumentation shapes the canal and removes infected dentin, it cannot completely eliminate microorganisms, tissue remnants, and debris from the intricate anatomy of the root canal system [1]. Anatomical complexities such as fins, isthmuses, lateral canals, and apical ramifications often remain inaccessible to endodontic instruments, making irrigation an indispensable component of chemo-mechanical preparation. The effectiveness of irrigation is determined not only by the irrigating solution but also by the method used to deliver and activate it within the canal [2].

Conventional syringe irrigation remains the most widely used technique because of its simplicity and ease of application. However, its ability to transport irrigants into the apical third and anatomically complex regions is limited by restricted fluid dynamics and the vapour lock effect [3,4]. To overcome these limitations, several irrigant activation systems have been introduced, including passive ultrasonic irrigation, sonic activation, air-sonic activation, and laser-assisted irrigation [1,5]. These systems enhance irrigant penetration by improving acoustic streaming, cavitation, and fluid movement, thereby facilitating better debridement and disinfection of areas that cannot be reached by endodontic instruments alone [5].

Mandibular molars, particularly their mesial canals, present a complex internal anatomy and are frequently associated with treatment challenges. Consequently, they provide an appropriate clinical model for evaluating the effectiveness of different irrigation activation techniques. Radiographic contrast media such as iohexol, owing to their radiopacity and physicochemical properties comparable to sodium hypochlorite, enable objective assessment of irrigant penetration using digital radiography [6]. Comparative clinical evidence regarding the penetration efficiency of newer activation systems, particularly diode laser-assisted irrigation and air-sonic activation, remains limited [7]. Therefore, evaluating these techniques under clinical conditions is important for identifying methods that optimize irrigant penetration and potentially improve the outcome of endodontic treatment.

The aim of the present study was to compare the depth of irrigant penetration achieved using conventional manual needle irrigation, passive ultrasonic irrigation, sonic irrigation, air-sonic irrigation (Pro-Agitator Tip System (PATS) Vario; Innovations Endo, Nashik, India), and diode laser-assisted irrigation in the mesial canals of mandibular molars. The objectives were to measure the depth of irrigant penetration with each irrigation technique using iohexol contrast medium and digital radiography, compare the penetration efficiency among the five irrigation systems, and determine the technique that provides the greatest apical penetration under clinical conditions.

## Materials and methods

This in vivo study was conducted in the Department of Conservative Dentistry and Endodontics, Institute of Dental Studies and Technologies, Modinagar, Uttar Pradesh, India. The study was carried out between January 2024 to June 2025 after obtaining approval from the Institutional Ethics Committee of the Institute of Dental Studies and Technologies (IDST/IEC/2022-25/02 dated April 25, 2023). Written informed consent was obtained from all participants prior to enrolment after explaining the nature, objectives, benefits, and possible risks of the study. The study was conducted in accordance with the ethical principles of the Declaration of Helsinki (2013 revision).

The sample size was estimated a priori using G*Power software version 3.1.9.7 (Heinrich Heine University Düsseldorf, Düsseldorf, Germany). Based on the findings of irrigant penetration depth into root canals in the reference study by Elgazzar et al. [8], considering an effect size (Cohen's f) of 0.52, a significance level (α) of 0.05, and a statistical power (1−β) of 80%, the minimum required sample size was calculated to be 50 teeth. Accordingly, 50 mandibular molars requiring primary root canal treatment were included in the study to ensure adequate statistical power for detecting differences among the irrigation techniques.

Patients aged between 18 and 50 years requiring primary endodontic treatment of mandibular first molars diagnosed with irreversible pulpitis and exhibiting two completely formed roots with curved mesial canals were considered eligible for inclusion. Teeth with previous endodontic treatment, open apices, root resorption, additional roots or canals, periodontal involvement, root fractures, calcified canals, or periapical lesions requiring surgical intervention were excluded. Patients with systemic conditions that could influence healing, including uncontrolled diabetes mellitus, renal disorders, thyroid disorders, sickle cell anaemia, or immunocompromised conditions, were also excluded from the study.

Following clinical and radiographic examination, all endodontic procedures were performed by a single experienced operator under local anaesthesia and rubber dam isolation. Standardized access cavity preparation was carried out, and the working length was determined using an electronic apex locator (Root ZX II; J. Morita Corp., Kyoto, Japan) and confirmed with digital intraoral radiography. Cleaning and shaping were completed using standardized instrumentation up to size 25/.04, while canals were irrigated between each instrument using 5.25% sodium hypochlorite (Prime Dental Products Pvt. Ltd., Mumbai, India) delivered through a Monoject syringe fitted with a 27-gauge side-vented irrigation needle (Cardinal Health Inc., Dublin, OH, USA). Following biomechanical preparation, a final rinse with normal saline was performed to eliminate residual sodium hypochlorite before assessment of irrigant penetration.

The irrigation activation technique employed for each tooth was selected according to the predefined observational study protocol without random allocation. Based on the activation method used during routine clinical treatment, the treated teeth were categorized into five equal observational groups (n = 10). Group 1 received conventional manual irrigation using the TruNatomy Irrigation Needle (Dentsply Sirona, Charlotte, NC, USA), a flexible 30-gauge side-vented polymer needle that delivered the irrigant 2 mm short of the working length using gentle manual pressure. Group 2 underwent passive ultrasonic irrigation using a non-cutting ultrasonic tip positioned 2 mm short of the working length and activated at the manufacturer's recommended power setting for 30 seconds to generate acoustic streaming and cavitation. Group 3 received sonic irrigation using the EndoActivator System (Dentsply Sirona) with a medium polymer tip (25/.04) operated at 10,000 cycles/min for 30 seconds, positioned 2 mm short of the working length to produce hydrodynamic agitation of the irrigant.

Group 4 received air-sonic activation using the PATS Vario, employing a polymer tip activated at the medium-frequency setting (approximately 4,000 Hz) for 30 seconds with the tip positioned 2 mm short of the working length to enhance three-dimensional irrigant movement. Group 5 received diode laser-assisted irrigation using the LX16 diode laser (Woodpecker Medical Instrument Co. Ltd., Guilin, China) equipped with a 200-µm optical fiber operated at a wavelength of 980 nm, 1.5 W continuous-wave mode, with the fiber inserted 2 mm short of the working length and activated for 20 seconds using gentle helicoidal withdrawal movements to promote photoacoustic streaming and irrigant activation.

For the evaluation of irrigant penetration, 1 mL of iohexol contrast medium (Omnipaque™ 300; GE HealthCare AS, Oslo, Norway) was introduced into the mesial canals using the respective irrigation activation technique, with the activation tip positioned approximately 2 mm short of the established working length. Immediately after contrast-medium application, standardized digital intraoral radiographs were obtained using the paralleling technique with a Snap-A-Ray® film holder (Dentsply Rinn, Elgin, IL, USA). The tooth was positioned with its long axis parallel to the image receptor, and the X-ray beam was directed perpendicular to the tooth and image receptor to minimize geometric distortion. The same exposure parameters and source-to-object/receptor positioning were maintained for all specimens. A radiographic image of a millimetre calibration scale was obtained using the same acquisition geometry to permit dimensional calibration of the digital images. The radiographs were imported into Sante DICOM Viewer (Santesoft Ltd., Athens, Greece), and the measurement scale was calibrated before assessment. The working length was used as the reference point, and a straight-line measurement was made from the radiographic endpoint corresponding to the established working length to the most apical extent of the radiopaque contrast medium within the mesial canal. The measurement was recorded in millimetres, with a smaller distance indicating greater apical penetration of the contrast medium. All measurements were performed by a calibrated examiner who was blinded to the irrigation technique. 

**Figure 1:**
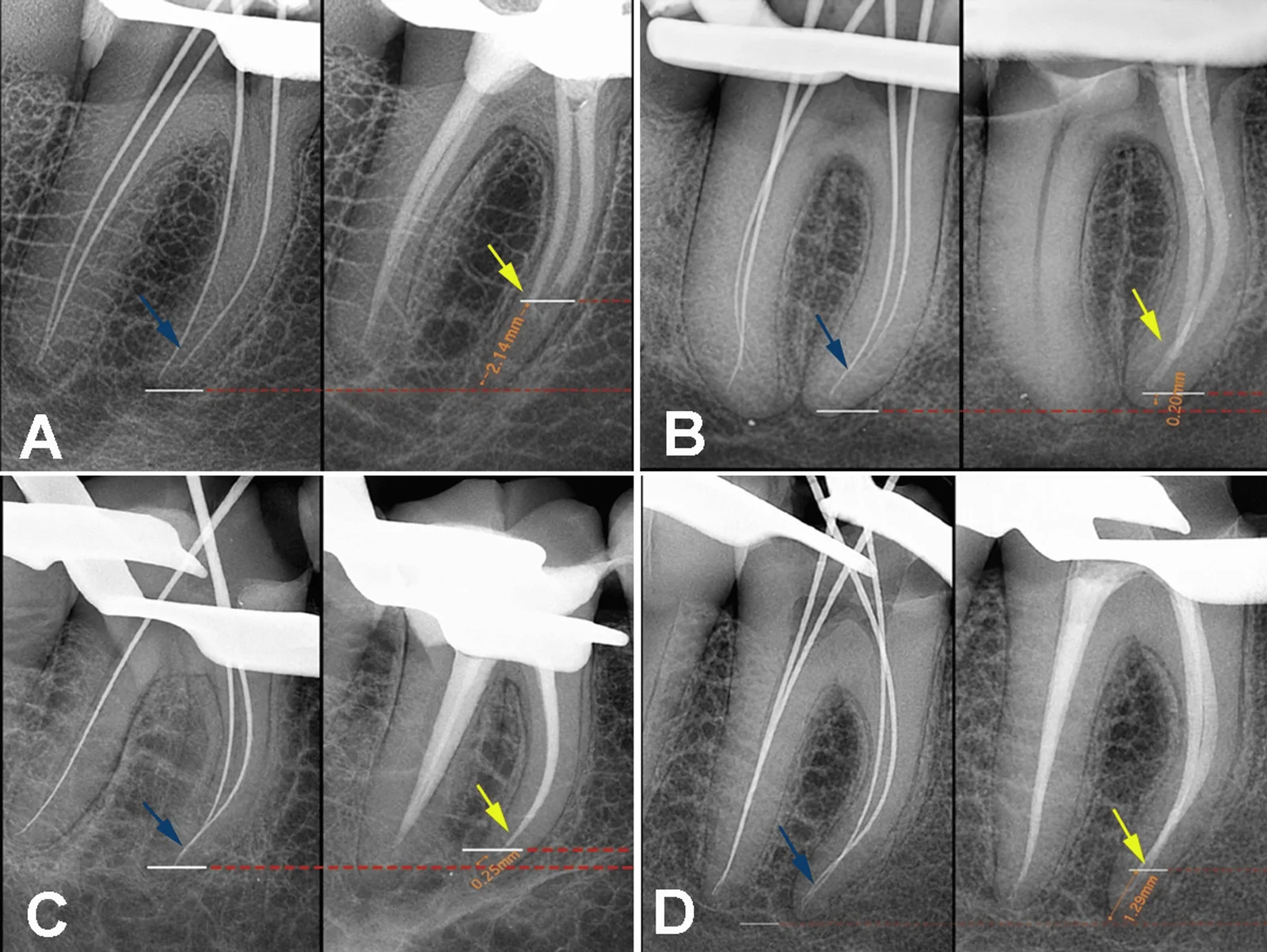
Intraoral periapical radiograph showed canal preparation, followed by irrigation with different techniques and infiltration with dye (A) Diode laser-assisted irrigation, (B) PATS air-sonic, (C) Sonic, (D) Ultrasonic. Blue arrow: Endodontic file in mandibular first molar canal (mesial canals). Yellow arrow: Dye infiltrate in mandibular first molar canal (mesial canals). PATS: Pro-Agitator Tip System

To minimize procedural variability, all included teeth underwent standardized access cavity preparation, working-length determination, and biomechanical preparation by a single experienced operator. The mesial canals were prepared using the same instrumentation protocol to an apical preparation size of 25/.04. Irrigation volume, needle/tip position, and activation duration were standardized according to the predefined protocol for each irrigation technique. All radiographic measurements were performed by a calibrated examiner who was not involved in the clinical procedures to minimize measurement bias. Each measurement was repeated after a two-week interval, and the mean of the two readings was considered for analysis. The primary outcome measure was the depth of irrigant penetration, expressed as the linear distance (mm) between the working length and the apical extent of the radiopaque contrast medium (Figure 1).

The collected data were analysed using IBM SPSS Statistics for Windows, Version 25.0 (IBM Corp., Armonk, NY, USA). Data normality was assessed using the Shapiro-Wilk test. Continuous variables were expressed as mean ± standard deviation and categorical data as frequency and percentage. Comparisons among the five irrigation groups were performed using one‑way analysis of variance (ANOVA) for age and the chi‑square (χ²) test for gender. Intergroup comparisons of irrigant penetration depth were performed using ANOVA followed by Tukey's honestly significant difference (HSD) post hoc test for pairwise comparisons when appropriate. Effect sizes were calculated using eta squared (η²) for the overall ANOVA and Cohen's d for pairwise comparisons. All statistical tests were two-tailed, and a p < 0.05 was considered statistically significant.

## Results

A total of 50 mandibular molars were included, with 10 teeth allocated to each irrigation activation technique. No significant differences were observed in age (p = 0.901) or sex (p = 0.891) among the groups (Table 1).

**Table 1:** Demographic analysis of the study groups. Data are presented as mean ± standard deviation (SD) for age and as number (percentage) for sex. Comparisons among the five irrigation groups were performed using one‑way analysis of variance (ANOVA) for age and the chi‑square (χ²) test for sex. PATS: Pro-Agitator Tip System

Parameter	Conventional manual irrigation (N = 10)	Passive ultrasonic irrigation (N = 10)	Sonic irrigation (EndoActivator) (N = 10)	PATS air-sonic irrigation (N = 10)	Diode laser-assisted irrigation (N = 10)	Test value	p-value
Age, (Mean ± SD)	26.45 ± 4.25	25.62 ± 4.52	25.78 ± 4.15	27.45 ± 5.12	26.28 ± 4.18	F = 0.261	0.901
Male, n(%)	5 (50)	6 (60)	4 (40)	5 (50)	4 (40)	χ² = 1.122	0.891
Female, n(%)	5 (50)	4 (40)	6 (60)	5 (50)	6 (60)		

One-way ANOVA demonstrated a statistically significant difference in irrigant penetration among the five irrigation activation techniques (p < 0.001, η² = 0.55) (Table 2). Diode laser-assisted irrigation showed the lowest mean dye distance from the working length (0.33 ± 0.26 mm), indicating the greatest apical penetration, followed by sonic irrigation (0.72 ± 0.42 mm), PATS air-sonic irrigation (0.87 ± 0.59 mm), and passive ultrasonic irrigation (1.23 ± 0.78 mm). Conventional manual irrigation demonstrated the highest mean dye distance (2.27 ± 0.88 mm), indicating the least effective irrigant penetration.

**Table 2:** Intergroup comparison of dye penetration depth (mm) among the irrigation activation techniques. Data are presented as mean ± standard deviation (SD) with 95% confidence interval (CI), Intergroup comparisons were performed using one-way analysis of variance (ANOVA), η² denotes eta-squared effect size and df indicates degrees of freedom, Penetration depth is expressed in millimetres (mm), a lower mean value indicates greater apical irrigant penetration, *p < 0.05 was considered statistically significant. PATS: Pro-Agitator Tip System

Groups	N	Mean ± SD (mm)	95% of CI of mean	F statistic (df)	p-value	Effect size (η²)
Conventional manual irrigation	10	2.27 ± 0.88	1.64-2.90	13.76 (4,45)	< 0.001*	0.55
Passive ultrasonic irrigation	10	1.23 ± 0.78	0.67-1.78			
Sonic irrigation (EndoActivator)	10	0.72 ± 0.42	0.42-1.02			
PATS air-sonic irrigation	10	0.87 ± 0.59	0.45-1.29			
Diode laser-assisted irrigation	10	0.33 ± 0.26	0.14-0.52			

Tukey's HSD post hoc analysis showed significantly greater dye distances with conventional manual irrigation than with diode laser-assisted irrigation (mean difference [MD] = 1.94 mm, p < 0.001), PATS air-sonic irrigation (MD = 1.40 mm, p < 0.001), sonic irrigation (MD = 1.55 mm, p < 0.001), and passive ultrasonic irrigation (MD = 1.04 mm, p = 0.008) (Table 3). Diode laser-assisted irrigation also demonstrated significantly greater apical penetration than passive ultrasonic irrigation (MD = −0.90 mm, p = 0.028), whereas no significant differences were observed between diode laser-assisted irrigation and PATS air-sonic irrigation or sonic irrigation (both p > 0.05).

**Table 3:** Pairwise comparison of dye penetration depth (mm) among the irrigation activation techniques using Tukey's honestly significant difference post hoc test. Data are presented as mean differences (row group − column group), Pairwise comparisons were performed using Tukey's honestly significant difference (HSD) post hoc test following a significant one-way ANOVA, Cohen's d was used to estimate effect size. Negative mean differences indicate lower dye penetration depth than the comparison group, *p < 0.05 was considered statistically significant. PATS: Pro-Agitator Tip System

Pairwise group		Mean difference (mm)	t-statistic	p-value	Effect size
Conventional manual irrigation	Diode laser-assisted irrigation	1.94	6.68	< 0.001*	2.99
Conventional manual irrigation	PATS air-sonic irrigation	1.40	4.82	< 0.001*	2.16
Conventional manual irrigation	Sonic irrigation (EndoActivator)	1.55	5.34	< 0.001*	2.39
Conventional manual irrigation	Passive ultrasonic irrigation	1.04	3.58	0.008*	1.60
Diode laser-assisted irrigation	PATS air-sonic irrigation	-0.54	-1.86	0.356	0.83
Diode laser-assisted irrigation	Sonic irrigation (EndoActivator)	-0.39	-1.34	0.678	0.60
Diode laser-assisted irrigation	Passive ultrasonic irrigation	-0.90	-3.10	0.028*	1.39
PATS air-sonic irrigation	Sonic irrigation (EndoActivator)	-0.15	-0.52	0.982	0.23
PATS air-sonic irrigation	Passive ultrasonic irrigation	-0.36	-1.24	0.740	0.55
Sonic irrigation (EndoActivator)	Passive ultrasonic irrigation	1.94	6.68	< 0.001*	2.99

## Discussion

The present in vivo study compared the depth of irrigant penetration achieved using manual irrigation, passive ultrasonic irrigation, sonic irrigation, PATS air-sonic irrigation, and diode laser-assisted irrigation in the mesial canals of mandibular first molars. The findings demonstrated a statistically significant difference among the irrigation techniques, with diode laser-assisted irrigation producing the greatest apical penetration, followed by sonic irrigation, PATS air-sonic irrigation, passive ultrasonic irrigation, and conventional manual irrigation. These findings emphasize that the method of irrigant activation plays a crucial role in improving irrigant delivery to the apical third of the root canal system.

Effective irrigation remains a cornerstone of successful endodontic treatment because mechanical instrumentation alone cannot adequately clean the complex anatomy of the root canal system [1]. Anatomical structures such as fins, isthmuses, lateral canals, and apical ramifications frequently harbor microorganisms and tissue remnants that are inaccessible to endodontic files [2]. Consequently, activation techniques that improve irrigant flow dynamics, acoustic streaming, and cavitation are expected to enhance penetration into these inaccessible regions, thereby improving canal disinfection [1].

The laser-assisted irrigation group demonstrated the smallest dye distance from the working length, indicating the greatest penetration efficiency [5]. This superior performance may be attributed to the photoacoustic and photomechanical effects generated by diode laser activation [9]. Rapid expansion and collapse of vapor bubbles within the irrigant create vigorous fluid movement, resulting in enhanced acoustic streaming and cavitation. These hydrodynamic phenomena enable the irrigant to overcome the vapor-lock effect within the apical third and facilitate penetration into complex canal irregularities [10]. In addition, the flexible laser fiber permits effective activation within curved canals while minimizing direct contact with dentinal walls [1]. Previous investigations evaluating laser-activated irrigation have similarly reported significantly greater irrigant penetration and superior canal cleanliness compared with conventional needle irrigation and several activation systems [5,9,11]. These observations support the present findings and reinforce the growing evidence that laser-assisted irrigation enhances the effectiveness of root canal disinfection.

Among the non-laser activation techniques, sonic irrigation demonstrated better penetration than passive ultrasonic irrigation and PATS, although the differences were not statistically significant [12]. Sonic activation generates hydrodynamic agitation through oscillation of flexible polymer tips, producing continuous fluid movement throughout the canal. The greater amplitude of oscillation achieved by sonic devices allows effective irrigant exchange, particularly within curved canals where unrestricted tip movement can be maintained [13]. Previous studies by Paragliola et al. [14], Caron et al. [15], and Pasqualini et al. [16] similarly reported improved irrigant penetration and debris removal with sonic activation compared with conventional syringe irrigation. The present findings are therefore consistent with earlier investigations demonstrating that sonic activation represents an effective alternative for enhancing irrigant distribution within the root canal system.

The PATS air-sonic irrigation system also demonstrated substantially better penetration than manual irrigation. PATS combines air-driven sonic activation with three-dimensional movement of a flexible polymer tip, generating effective acoustic streaming without the increased risk of metallic tip fracture [17]. Although relatively few clinical studies have evaluated this newer activation system, available evidence suggests that PATS improves irrigant circulation within the canal and facilitates better cleaning of anatomically complex regions [17,18]. The comparable performance of PATS and sonic activation observed in the present study indicates that both systems provide clinically effective irrigant activation while maintaining procedural simplicity.

Passive ultrasonic irrigation produced intermediate penetration values. Ultrasonic activation is known to generate high-frequency oscillations and acoustic streaming capable of disrupting debris and improving irrigant exchange [19]. However, the efficiency of ultrasonic activation is influenced by unrestricted oscillation of the ultrasonic tip within the canal. In curved or narrow canals, contact between the ultrasonic tip and dentinal walls may dampen oscillation, reducing the magnitude of acoustic streaming and cavitation. This phenomenon may explain why ultrasonic irrigation, although significantly superior to manual irrigation, demonstrated lower penetration efficiency than laser-assisted irrigation in the present investigation. Similar observations have been reported by Mozo et al. [19] and Llena et al. [20], who concluded that although passive ultrasonic irrigation enhances canal cleanliness, its effectiveness depends on adequate canal enlargement and unrestricted file oscillation.

Conventional manual irrigation demonstrated the poorest irrigant penetration among all groups. Needle irrigation primarily relies on positive-pressure delivery of the irrigant, which produces limited fluid replacement beyond the needle tip. Furthermore, the vapor-lock phenomenon in the apical third restricts irrigant exchange and limits penetration into lateral canals and isthmuses [1]. These limitations explain the significantly greater dye distance observed in the manual irrigation group. Comparable findings have been consistently reported in previous investigations, including the study by Elgazzar et al. [8], which demonstrated significantly lower penetration efficiency with conventional syringe irrigation compared with sonic activation techniques. The present results further support the concept that irrigation activation is essential for achieving effective irrigant penetration within the apical region.

Clinical implications

The findings of this study suggest that activation of irrigants should be considered an integral component of contemporary endodontic treatment rather than relying solely on conventional syringe irrigation. Diode laser-assisted irrigation demonstrated the greatest penetration efficiency and may provide superior debridement of anatomically complex regions, potentially improving microbial reduction and treatment outcomes. Where laser systems are unavailable, sonic activation and PATS air-sonic irrigation may serve as effective alternatives because they significantly enhance irrigant penetration compared with manual irrigation while remaining relatively simple to incorporate into routine clinical practice.

Limitations

The present study has certain limitations. The sample size was relatively small and the study was conducted at a single center, which may limit the generalizability of the findings. Irrigant penetration was evaluated using a radiographic contrast medium as an indirect measure and did not assess actual bacterial reduction or long-term treatment success. Only mandibular molars were included, and therefore the results may not be directly applicable to teeth with different canal morphologies. Although canal preparation was standardized to size 25/.04, the preoperative canal volume and three-dimensional canal morphology were not independently quantified. Therefore, residual anatomical variability among the mesial canals may have influenced irrigant penetration. Future multicenter clinical studies with larger sample sizes, three-dimensional imaging techniques, microbiological assessment, and long-term clinical follow-up are recommended to validate the present findings and determine their influence on endodontic treatment outcomes.

## Conclusions

Within the limitations of this in vivo study, the method of irrigant activation significantly influenced apical irrigant penetration in mandibular molars. Diode laser-assisted irrigation demonstrated the greatest penetration efficiency, followed by sonic irrigation, PATS air-sonic irrigation, passive ultrasonic irrigation, and conventional manual irrigation. Conventional syringe irrigation showed the least effective apical penetration. These findings suggest that activation techniques, particularly diode laser-assisted irrigation, can enhance irrigant delivery into the apical region and may improve the effectiveness of root canal disinfection. Further multicenter studies with larger sample sizes and long-term clinical evaluation are recommended to confirm these findings.
